# Biases in preferences for sequences of outcomes in monkeys^☆^

**DOI:** 10.1016/j.cognition.2013.11.012

**Published:** 2014-03

**Authors:** Tommy C. Blanchard, Lauren S. Wolfe, Ivo Vlaev, Joel S. Winston, Benjamin Y. Hayden

**Affiliations:** a Department of Brain and Cognitive Sciences, University of Rochester, Meliora Hall, Rochester, NY 14627, United States; b Center for Visual Science, University of Rochester, Meliora Hall, Rochester, NY 14627, United States; c Centre for Health Policy, South Kensington Campus, London SW7 2AZ, United Kingdom; d Department of Surgery and Cancer Imperial College London, South Kensington Campus, London SW7 2AZ, United Kingdom; e UCL Institute of Cognitive Neuroscience, University College London, 12 Queen Square, London WC1N 3BG, United Kingdom; f Wellcome Trust Centre for Neuroimaging, University College London, 12 Queen Square, London WC1N 3BG, United Kingdom; g Department of Neurology, Northwestern University, 303 East Chicago Avenue, Ward 10-185, Chicago, IL 60611, United States

**Keywords:** Heuristics, Biases, Intertemporal choice, Peak-end, Macaque

## Abstract

Movies, vacations, and meals are all examples of events composed of a sequence of smaller events. How do we go from our evaluations of each scene in a movie to an evaluation of the sequence as a whole? In theory, we should simply average the values of the individual events. In practice, however, we are biased towards sequences where each element tends to be better than the previous, where the last value is large, and we overweight the best (or worst) part of the sequence. To study how general these biases are we examined monkeys’ preferences for sequences of rewards in a novel *reward repeat* task. Monkeys were first given a sequence of rewards and then chose between repeating the sequence or receiving a standard comparator sequence. We found that, like humans, monkeys overweight events that happen later in a sequence, so much so that adding a small reward to the end of a sequence can paradoxically reduce its value. Monkeys were also biased towards sequences with large peak values (the highest value in the sequence), but only following a working memory challenge, suggesting that this preference may be driven by memory limitations. These results demonstrate the cross-species nature of biases in preferences for sequences of outcomes. In addition, monkeys’ consistent preference for sequences in which large values occur later challenges the generality of discounting models of intertemporal choice in animals.

## Highlights

• A new reward-repeat task that allows monkeys to report valuations of sequences.
• Predictable biases in monkeys’ evaluations of sequences similar to human biases.
• Monkeys are biased towards sequences with larger values near end.
• Peak-bias evoked by weak working-memory challenge.

## 1 Introduction

We must often evaluate experiences that occur over extended periods of time and involve a mix of reward intensities and valences (Loewenstein & Prelec, 1993). For example, how much did we enjoy a specific two-hour movie, three-course meal, or seven-day vacation? And how much would we pay for another similar sequence? To make these evaluations, we must mentally combine the utilities of multiple individual moments into a single value. In theory we should just sum the experienced utilities of the constituent events – the order of events occur should not matter. How much we feel we have enjoyed a movie should just be a function of how much we enjoyed each scene individually. However, human and animal decision-makers typically discount rewards as an increasing function of delay (Rachlin, 2004). One would expect, then, that we would prefer sequences with the highest valued events early (Frederick, Loewenstein, & O’Donoghue, 2002; Loewenstein & Prelec, 1993), meaning a movie with a strong start would be preferred to one with a strong ending.

Common sense dictates that the overall utility of an experience is the sum (or average) utility of its components. Discounting models predict that, because we prefer rewards sooner rather than later, we should invariably prefer declining sequences to improving ones that are matched for average value. Contrary to both of these, humans often prefer sequences to increase in value over time (Ariely, 1998; Chapman, 1996; Chapman, 2000; Frank & Hutchens, 1993; Hsee, Abelson, & Salovey, 1991; Hsee & Abelson, 1991; Loewenstein & Prelec, 1993; Loewenstein & Sicherman, 1991; Prelec & Loewenstein, 1991; Ross & Simonson, 1991). Closely related biases motivate us to value sequences in which the more positive events occurred near the end (the *end bias*), sequences with greater peak intensities regardless of when they occur (the *peak bias*), and sequences with increasing reward intensity (the *trend bias*) (Kahneman, Fredrickson, Schreiber, & Redelmeier, 1993; Loewenstein & Prelec, 1993; Redelmeier & Kahneman, 1996). That is, we would expect that people overweight the best scene and ending of a movie when reporting how much they enjoyed the film. Together, these biases are a defining feature of our intertemporal preferences. These preference patterns apply to positive and aversive domains, have been confirmed in field studies, and may be harnessed to improve health and welfare (Clark & Georgellis, 2004; Do, Rupert, & Wolford, 2008; Kahneman, 1999; Redelmeier & Kahneman, 1996; Redelmeier, Katz, & Kahneman, 2003). So robust are these effects that merely reframing rewards as being part of a sequence rather than independent events can cause a switch in preference from decreasing towards increasing sequences (Loewenstein & Prelec, 1993). Together, these results pose a major challenge to standard theories about temporal allocation of rewards and suggest that psychological factors can overwhelm discounting preferences in intertemporal choice (Frederick, Loewenstein, & O’Donoghue, 2002).

Understanding animal economic preferences provides an important point of comparison with human economic preferences (Real, 1991; Stevens & Stephens, 2010). Studies of animals economic preferences in the laboratory have provided a great deal of information about the cognitive processes they use to make decisions, giving us insight into the mental lives of animals (Brosnan & de Waal, 2003; Brosnan et al., 2007; Chen, Lakshminarayanan, & Santos, 2006; Egan, Bloom, & Santos, 2010). We know almost nothing about how animals evaluate sequences of rewards (but see Xu, Knight, & Kralik, 2011). The well-established steep discounting observed across taxa would predict that animals strongly prefer decreasing sequences to flat ones and flat ones to increasing ones.

We studied the preferences of three rhesus monkeys in a simple sequence preference task. Rhesus monkeys offer an ideal model organism for studying intertemporal preferences – their psychology is well studied, they are flexible learners that do not readily fall into behavioral stereotypies, and they have time preferences that are similar to those of other animals (Glimcher, Kable, & Louie, 2007; Kim, Hwang, & Lee, 2008; Stevens, Rosati, Ross, & Hauser, 2005). On each trial of our novel *reward repeat* task, monkeys were given a sequence of five rewards and then offered a choice between repeating that sequence or obtaining a well-learned comparator sequence consisting of five repeats of a single value (either [2.2 2.2 2.2 2.2 2.2], [3 3 3 3 3], [3.4 3.4 3.4 3.4 3.4], or [4 4 4 4 4]). We then estimated a subjective value for each probe sequence by fitting a preference function.

We found that monkeys assign more decision weight to events later in the sequence. Indeed, the addition of a small reward at the end of a sequence can, paradoxically, reduce its value (cf. Kahneman et al., 1993; Schreiber & Kahneman, 2000). These results are reminiscent of those observed in humans and suggest that similar heuristics are employed by humans and monkeys in guiding choices over extended sequences. Unlike humans, monkeys did not prefer sequences with large peaks in the standard version of our task, although we induced a peak bias in monkeys by adding a weak working memory challenge (a four second delay preceding choice). Collectively, these results highlight the potential importance of memory in driving preferences and challenge discounting models of animal intertemporal preferences.

## 2 Methods

### 2.1 Behavioral task

Trials were randomly chosen from a larger set of possible sequences and interleaved. We collected about twice as much data from subject H as from subjects J and K. The amount of data collected was determined by subject and experimenter availability and was not in any way determined by examining data. No subjects were excluded from the study. Our computer monitor had a 1024 × 768 resolution and was placed 144.8 cm (57 in.) in front of the subjects.

**Fig. 1:**
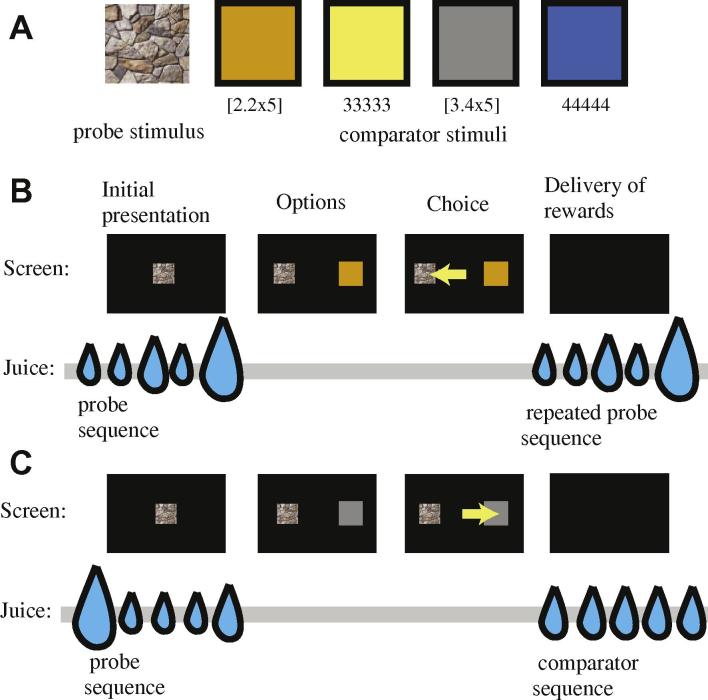
Schematic of the design of the ***reward-repeat task***. (**A)** Stimuli used. On each trial, subjects chose between a probe stimulus (left image) and one of four different standard stimuli (four colored squares, right). Reward units are multiples of 10 μL. (**B** and **C)** Timeline of task, two examples. On each trial, subjects were initially presented with a sequence of 5 fluid rewards; each element was separated by 0.5 s; the probe stimulus image appeared centrally on the computer monitor during this time. Then, following a brief delay (0.5 s), the probe stimulus and one standard stimulus appeared on the left and right of the fixation spot (sides were randomized on each trial). (B) Selection of the probe led to a repeat of the probe sequence and (C) selection of the standard led to the appropriate standard sequence. Inter-trial interval was 1 s.

Each trial of the task consisted of three steps:

*Step 1, probe reward:* The probe cue appeared (a photograph of some rocks, see Fig. 1). After 500 ms, a sequence of five fluid rewards was given, each separated from the next by 500 ms. The duration of the rewards ranged from 20 ms to 80 ms. The time required to give the reward did not slow down the delivery of the next reward. The identity of the rewards varied with task condition (see below). When the number zero appears in a sequence, this indicates no reward was given but a 500 ms delay still occurred. In the one four-step sequence [2 2 2 8], no delay was imposed at the end. In the ***working memory challenge*** variant of the task, a four-second delay was added at the end of the probe reward period and before the choice; the monitor was kept blank during this time. Note that, for ease of description, values are normalized to a standard value of 1 = 0.01 mL.

***Step 2, choice:*** Following a 500 ms delay, two targets appeared, centered 16.5 degrees of visual angle to the left and right of the central spot. The **probe** stimulus consisted of the same neutral photographic stimulus that appeared in Step 1 (the picture of rocks). The **comparator** stimulus consisted of a solid colored vertically oriented rectangle (80 × 300 pixels). The comparator was one of four colors (orange, yellow, gray, or blue) indicating the associated reward size. Choice of the comparator offered one a sequence of five repeats of the same size (2.2, 3.0, 3.4, and 4.0, respectively). The side on which the probe and comparator were shown was chosen randomly on each trial. Monkeys made their choice by shifting gaze to the chosen target.

***Step 3, reward:*** 500 ms following the monkeys’ choice, the chosen reward was given. Choice of the probe offered an immediate repeat of the sequence that was given in Step 1. After that, an inter-trial interval of 1 s occurred.

### 2.2 Training

All three subjects were previously extensively trained to perform simple decision making tasks involving making saccades to targets for rewards. Two of the subjects (H and J) performed, among other tasks, intertemporal choice tasks; the other subject (K) did not. All three subjects were familiar with the mappings between colors and reward size, which are maintained across all tasks within the lab. Training involved a stepwise series of tasks building towards the standard task. Subjects were required to be trained for at least 2 weeks, and were not allowed to begin data collection until they were able to perform a simplified version of the task at 75% accuracy for three days in a row. (This simplified version involved four uniform sequences equal to the standard sequences; accuracy in this version was choosing the option, probe or comparator, with the larger total reward.) In practice, all three subjects took about two weeks to meet this criterion.

### 2.3 Statistics

Matlab (Mathworks, Natick, MA) was used for all computations. The point of subjective equivalence (PSE) was computed by fitting a sigmoidal curve to the preference data and then computing the point where this curve crossed the indifference (i.e. 50% choice) line (similar methods were used in Hayden, Parikh, Deaner, & Platt, 2007 and Hayden et al., 2007). Standard errors were estimated using a jackknife method (as in Hayden et al., 2007). Specifically, we repeated the calculation of PSE on a subset of 95% of the data, randomly selected. Then we repeated this procedure 20 times, using different random sets (without replacement) and used these 20 estimates to compute the standard error. The jackknife standard deviation was defined as the standard error multiplied by the square root of 20.

**Fig. 2:**
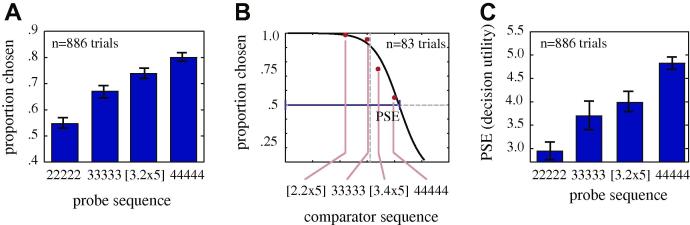
Basic preference data for all three monkeys for flat sequences. (**A)** Average proportion of trials on which monkeys chose each of four flat sequences (i.e. sequences with five repeats of the same value). Data are averaged across the four standard stimuli. Monkeys chose larger valued sequences more often, confirming that their behavior was sensible. Error bars indicate standard error. (**B)** Plot of proportion chosen for the probe sequence [3.2 3.2 3.2 3.2 3.2] as a function of the four different *comparator sequences* (red dots). Monkeys were more likely to choose higher valued comparators. Black line indicates best-fit sigmoidal curve. Blue line highlights the point of subjective equivalence (PSE), our model estimate of the value at which the monkey was indifferent to the probe and comparator. The PSE provides an estimate of the decision utility assigned to the probe sequence. (**C**) Plot of the PSE for each of the four flat probe sequences shown in panel A. PSE rose with probe value and was in all cases about ∼0.8 fluid units greater than the comparator value. Error bars indicate standard error and are calculated by a jackknife procedure (see Section 2.3). (For interpretation of the references to color in this figure legend, the reader is referred to the web version of this article.)

**Fig. 3:**
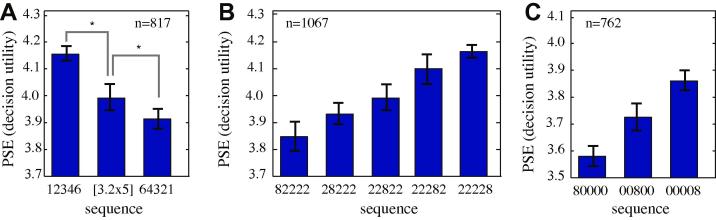
Preferences of three monkeys for ordered sequences. (**A**) Monkeys assign greater value to increasing sequences than to flat sequences or decreasing sequences, even when total intake is matched. (**B**) Preference for large value at the end of the sequence is maintained even when small values are zeros (no reward). (**C**) Monkeys assign greater value to sequences with a single large value the later it is in the sequence. In all cases, error bars indicate standard error as computed by jackknife (see Section 2.3).

Computing whether ordered variables have a significant effect on computed variables like PSE presents a special problem for analysis because we cannot use simple linear regression (we are referring here to the analyses shown in Figs. 2C and 3A–C). We therefore devised a novel statistical technique using a bootstrap methodology, which we refer to above as a ***bootstrap permutation test***. We first performed a random statistical resampling of the dataset (with replacement) to generate a resampled version of the dataset (of the same size as the original). We then computed the PSE for each of the elements of this resampled distribution and then calculated the slope of the best-fit line through these points. We then repeated this process 20,000 times and counted the proportion of times the slope crossed over zero. We multiplied this proportion by two (because we wanted to get a two-tailed *t*-test) to estimate the *p*-value for the claim.

### 2.4 Subjects

All procedures were approved by the University of Rochester Institutional Animal Care and Use Committee and were designed and conducted in compliance with the Public Health Service’s Guide for the Care and Use of Animals. Three male rhesus monkeys (*Macaca mulatta*) served as subjects. Initially, each animal was provided with a small mount to facilitate head positioning using standard techniques (Blanchard, Pearson, & Hayden, 2013). Subjects were habituated to laboratory conditions and trained to perform decision tasks for liquid reward. Standard reinforcement training was used with only positive fluid rewards; punishment was never used, nor was aversive conditioning.

## 3 Results

On each trial of our novel ***reward repeat task*** (Fig. 1), monkeys first experienced a sequence of five liquid rewards (*probe sequence*) and then immediately chose between two options: (a) repeating the probe sequence of rewards, (b) taking one of four standard *comparator sequences*. The probe sequence is chosen randomly each trial from a larger set of possible sequences (the larger set of sequences is determined by the experimental condition). For ease of description in this manuscript, all juice volumes are normalized to a standard value of 1 aliquot = 0.01 mL. Comparator sequences consisted of five repeats of a single value (either 2.2, 3, 3.4, or 4), were well-learned during training, were explicitly cued by color, and varied randomly each trial. By fitting a sigmoidal curve to preference as a function of probe sequence value, we estimated a subjective value for each probe sequence (Hayden, Heilbronner, & Platt, 2010; Hayden et al., 2007) (see Section 2.3).

### 3.1 Preferences for uniform sequences confirm basic behavioral coherence

We first examined monkeys’ evaluation of probe sequences consisting of five identical options. Fig. 2A shows the average frequency of choices for each sequence ([2 2 2 2 2], [3.2 3.2 3.2 3.2 3.2], [3.6 3.6 3.6 3.6 3.6], and [4 4 4 4 4]) against the four comparator sequences [2.2 2.2 2.2 2.2 2.2], [3 3 3 3 3], [3.4 3.4 3.4 3.4 3.4], and [4 4 4 4 4]. Monkeys choose the greater valued sequence more often than the less valued ones (regression of preference against probe value, *β* = 0.10, *p *= 0.0002). The same patterns are observed for each monkey individually (subject H: *β* = 0.11, *p *= 0.02; subject J: *β* = 0.09, *p *= 0.01; subject K: *β* = 0.07, *p *= 0.04). We next examined the influence of session by treating it as a random effect in a multi-variable ANOVA (subject × sequence × session). We found no influence of session (*p *= 0.1374, DF = 36, sum of squares = 7.306, *F* = 1.26) or of subject (*p *= 0.173, DF = 2, sum of squares = 0.549, *F* = 1.76), although we did find a significant effect of sequence (*p *= 0.0003, DF = 3, sum of squares = 3.003, *F* = 6.41).

To determine the value placed on each sequence, we next estimated the point of subjective equivalence (PSE)for each sequence (illustrated in Fig. 2B). To do so, we separated choices by the value of the standard comparator. Data for the probe sequence [3.2 3.2 3.2 3.2 3.2] are shown in Fig. 2B; the four comparators appear on the *x*-axis. We then fit the data to a sigmoidal curve. We defined the PSE as the point where this sigmoidal curve crosses the indifference line (i.e. 50% choice of probe and 50% choice of standard), indicating equal valuation of the two options. The PSE provides an estimate of the subjective value placed on the sequence of options in terms of the value of the standard comparators. The PSE for this sequence was 4.0 (±0.43 standard error), indicating that monkeys treated this sequence as if it had a value equivalent to [4 4 4 4 4].

The difference between this decision value for a sequence and its actual value, 0.8 (for each element in the sequence), provides a measure of monkeys’ intrinsic preference for the probe option vs. the comparator. This *probe premium* was roughly constant for all four uniform sequences used in Experiment 1 and for each of the three monkeys. Indeed, we found no relationship between the probe premium and probe value (we used a novel bootstrap slope test for this analysis, see Section 2.3; we abbreviate slope as *m*, *m* = 0.023, *p *= 0.55). Our data do not provide any information about why the probe sequences were preferred to the comparators; we speculate that this difference may reflect a preference for variable, novel, or informative options (Bromberg-Martin & Hikosaka, 2009; Hayden, Heilbronner, Nair, & Platt, 2008; Heilbronner & Hayden, 2013).

Fig. 2C shows the PSE for all four probe sequences used in Experiment 1, [2 2 2 2 2], [3.2 3.2 3.2 3.2 3.2], [3.6 3.6 3.6 3.6 3.6], and [4 4 4 4 4]. Not surprisingly, we found a clear monotonic relationship between probe value and decision value. We observed a significant positive relationship between probe value and PSE for the group of three monkeys and for each monkey individually (using the bootstrap slope test). Specifically, *m* = 0.9 (units for slope are aliquots per trial divided by a categorical dummy variable), *p *< 0.0001 for the group of monkeys (subject H: *m* = 1.1, *p *< 0.0001; subject J: *m* = 0.7, *p *= 0.0009; subject K: *m* = 0.55, *p *= 0.033). Together these data indicate that monkeys have no trouble understanding the task. These data also indicate that monkeys have well-behaved, systematic preferences over the options and a clear preference for the probe over the comparator option.

### 3.2 Monkeys prefer sequences with large rewards at the end

We next examined how monkeys evaluate increasing, flat, and decreasing sequences (Fig. 3A). We first examined the rising sequence [1 2 3 4 6], the flat sequence [3.2 3.2 3.2 3.2 3.2], and the falling sequence [6 4 3 2 1]. These three sequences, as well as most used in this study, have the same total value (16). We found a clear preference for the increasing sequence (bootstrap *t*-test, *p *< 0.0001 for the group, *p *< 0.0001 for H, *p *< 0.001 for J and K) and a clear preference for the flat sequence over the decreasing one (*p *< 0.008 for the group, and *p *= 0.012 for H, *p *= 0.015 for J and *p *= 0.029 for K). Thus monkeys prefer sequences to increase in value rather than decrease. These preferences are inconsistent with discounting theories, which predict preference for decreasing sequences, and are reminiscent of human preferences for increasing sequences (Ariely, 1998; Loewenstein & Prelec, 1993).

We next examined the influence of session by treating it as a random effect in a multi-variable ANOVA (subject × sequence × session) on the raw preference data (not the PSE fit data). We found no influence of session (*p *= 0.3805, DF = 36, sum of squares = 7.533, *F* = 1.06) or of subject (*p *= 0.4791, DF = 2, sum of squares = 0.284, *F* = 0.74).

We then examined the effect of position on preference (Fig. 3B). We tested five reward sequences [8 2 2 2 2], [2 8 2 2 2], [2 2 8 2 2], [2 2 2 8 2], and [2 2 2 2 8]. These sequences are identical except for the temporal position of the large reward. We found a clear and roughly linear increase in value for sequences with later positioning of the large reward. Specifically we found values of *m* = 0.08 (units for slope are aliquots per trial divided by a categorical dummy variable), *p *< 0.0001 for the group (subject H: *m* = 0.083, *p *< 0.0001; subject J: *m* = 0.096, *p *= 0.01; subject K: *m* = 0.062, *p *= 0.01). These data confirm that the order in which options are presented matters to monkeys in determining preference, and that larger rewards presented later in the sequence are preferred to larger rewards sooner. This pattern of preferences is consistent with the preference for increasing sequences observed in humans and is somewhat consistent with the end bias (Ariely, 1998; Kahneman et al., 1993; Schreiber & Kahneman, 2000).

We next examined the possible confounding factor of session and/or subject by treating them as a random effect in a multi-variable ANOVA (subject × sequence × session) on the raw preference data (not the PSE fit data). We found no influence of session (*p *= 0.6111, DF = 36, sum of squares = 6.43, *F* = 0.92) or of subject (*p *= 0.8103, DF = 2, sum of squares = 0.08, *F* = 0.21). We thus infer that sequence order was the critical factor.

Monkeys’ valuations of sequences appear to contradict the *discounting hypothesis*, that monkeys steeply discount future rewards over the course of a few seconds (Rachlin, 2004). This hypothesis predicts that monkeys should inevitably prefer decreasing sequences (Frederick, Loewenstein, & O’Donoghue, 2002). However, the intertemporal choice tasks that are used to support the discounting hypothesis generally use single rewards. Thus it is possible that monkeys show anti-discounting behavior in our reward repeat task because of some bias caused by multiple rewards. To test this idea, we examined valuations for three sequences, [8 0 0 0 0], [0 0 8 0 0], and [0 0 0 0 8] (Fig. 3C). Zeros here correspond to half-second periods with no reward (or any other novel cue). Again, we found a clear pattern of preferences for large reward later. Specifically, we found a significant effect of position on value using the bootstrap permutation test, *m* = 0.12, *p *< 0.0004 for the group (subject H: *p *= 0.006; subject J: *p *= 0.04; subject K: *p *= 0.011). Interestingly, the probe premium for these sequences (roughly 2.0 across animals and conditions) was greater than that observed for other, more valuable sequences (about 0.8, as noted above). Within the hyperbolic discounting framework, the present results can only be accounted for by a negative discount factor k (that is, rewards are more valuable when delayed than when immediate). Because our monkeys have positive ks in standard discounting tasks (Blanchard et al., 2013), we surmise that the discounting parameter measured by intertemporal choice tasks may have poor external validity, as suggested by earlier work (Bateson & Kacelnik, 1996; Blanchard et al., 2013; Pavlic & Passino, 2010; Pearson, Hayden, & Platt, 2010; Stephens & Anderson, 2001).

We next examined the possible confounding factor of session and/or subject by treating them as a random effect in a multi-variable ANOVA (subject × sequence × session) on the raw preference data (not the PSE fit data). We found no influence of session (*p *= 0.9645 DF = 36, sum of squares = 5.002, *F* = 0.62) or of subject (*p *= 0.733, DF = 2, sum of squares = 0.136, *F* = 0.31).

### 3.3 Paradoxical effect of adding a small reward

Human studies have shown that adding a punisher to the end of a sequence of negative events can, paradoxically, increase preference for that sequence if the punisher is less aversive than earlier events (Schreiber & Kahneman, 2000). For example, holding one’s hand in a bucket of 14° water for one minute and then an additional 30 s while the water is raised to 15° (still unpleasant but less so) is generally preferred to the one minute 14° water event alone (Kahneman et al., 1993). Similar effects have been observed with positive experiences (Fredrickson & Kahneman, 1993; Shizgal, 1999). We therefore wondered whether adding a small reward to the end of a sequence might decrease preference for it.

**Fig. 4:**
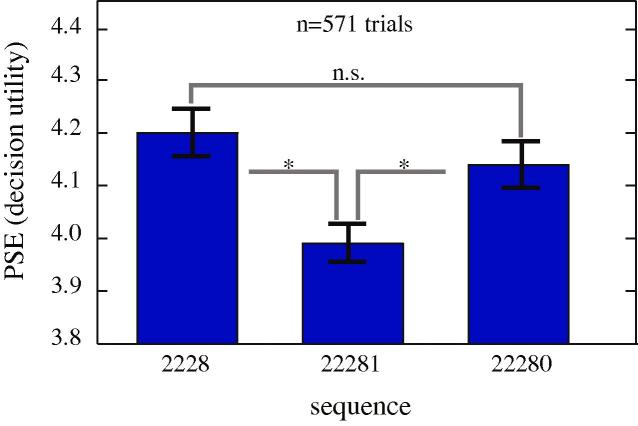
Adding a small reward at the end of a sequence can, paradoxically, reduce its subjective value. This effect does not appear to reflect an attempt to rate maximize, as including a delay (zero) does not significantly reduce preference.

We thus next compared valuations for the sequences [2 2 2 8] and [2 2 2 8 1] (Fig. 4). Because the first sequence had one fewer element and was therefore 500 ms shorter, we were concerned that monkeys might choose it in order to hasten the beginning of the next trial and increase reward intake rate (Blanchard et al., 2013). We therefore added another condition with the same timing as the [2 2 2 8 1] sequence, [2 2 2 8 0]. We found that monkeys preferred [2 2 2 8] and [2 2 2 8 0] to [2 2 2 8 1], despite the fact that the sequence [2 2 2 8 1] offers more total reward (15 vs. 14). These effects are significant (bootstrap permutation test, *p *= 0.004 for the subjects together, *p *< 0.05 for each of the three subjects individually). We found no significant difference in preference between [2 2 2 8] and [2 2 2 8 0], suggesting that timing does not contribute strongly to preferences in this condition (bootstrap permutation test, *p *= 0.18 for the group of three subjects, *p *> 0.05 for each of the individual subjects).

We can also look at the sequence [2 2 2 8 2] from the earlier section. The PSE for this sequence (4.11) was significantly lower than the PSE for [2 2 2 8] (4.2, bootstrap permutation test, *p *= 0.044). These results indicate that even increasing a sequence’s value by 2 (that is, by about 14% of the value of the original sequence) is not enough to overcome the reduction in preference induced by ending with a lower value in this context.

It is not clear why the sequence [2 2 2 8 0] is not treated as a sequence with a very low end value. We conjecture that the single zero at the end of [2 2 2 8 0] may be more readily ignored than the two and four zeroes at the end of the other one-element sequences.

### 3.4 Later elements in the sequence have greater influence on evaluations

Taken together, these data indicate that, within the context of this task, monkeys prefer sequences with increasing values and that they are particularly motivated by large values at the end of a sequence. One possible explanation for these data is that monkeys place more weight on items later in the sequence. To test this idea, we next measured valuations in a ***random sequence*** variant of the reward repeat task. In this version of the task, every element of every sequence on every trial was chosen at random by the computer. Rewards in each of the five steps of the sequence were 1, 2, 3, 4, 5, or 6. (Random selection was made with replacement, so it was possible for the same element to reappear, and sequence average values were not constrained).

**Fig. 5:**
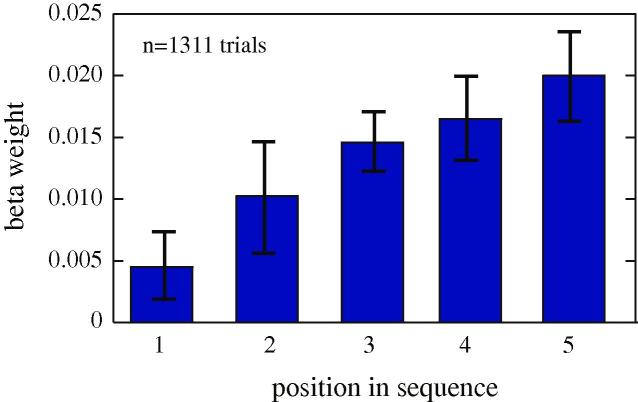
Impact of position in sequence on upcoming valuation. Results of regression of position against likelihood of choosing in the random sequence variant of the task. Stimuli later in the sequence exhibit a stronger effect on choice. Error bars indicate standard error (see Section 2.3).

We regressed preferences (as defined by PSE, see Section 2.3) against reward value in each position, which were independent of each other (Fig. 5). We found that monkeys’ regression coefficients increase roughly linearly with step number. Specifically, we found a significant increase in regression coefficient with step number for the group (bootstrap permutation test, *p *< 0.0001) and for each monkey individually (*p *< 0.0001 for H, *p *= 0.006 for J and *p *= 0.044 for K). These effects indicate that the size of the reward later in the sequence has a greater effect on preference than the size of the reward earlier in the sequence.

We next examined the possible confounding factor of session and/or subject by treating them as a random effect in a multi-variable ANOVA (subject × sequence × session) on the raw preference data (not the PSE fit data). We found no influence of session (*p *= 0.7476 DF = 6, sum of squares = 0.673, *F* = 0.58) or of subject (*p *= 0.4171, DF = 2, sum of squares = 0.339, *F* = 0.88).

### 3.5 Peak bias and working memory challenge

**Fig. 6:**
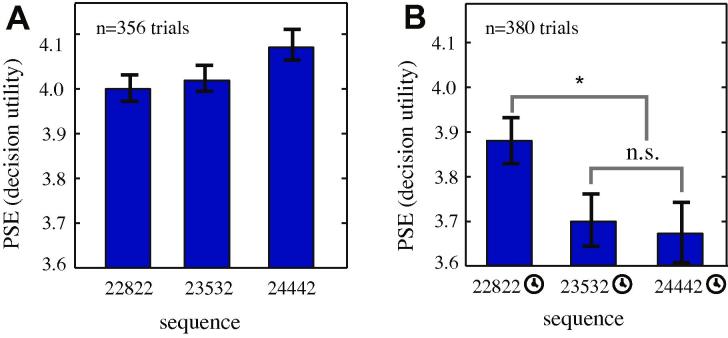
Influence of peak value on preference with and without working memory challenge. (**A**) Plot of value placed on each of three sequences with same total value but different peak. Subjects prefer sequences with flatter distribution of values. (**B**) Introducing a weak working memory challenge (a 4 s delay after initial presentation of stimuli and before choice) induces a preference for peaked sequences. Error bars indicate standard error, as calculated by a jackknife procedure (see Section 2.3).

Besides the end bias and preference for improving sequences, humans have a pronounced ***peak bias***. That is, we often prefer sequences with a single large value to ones with a higher total value but a smaller peak (Do et al., 2008). We observed no evidence of peak biases in our data. Fig. 6A shows the relative preferences for three sequences with the same average value but different peak levels: [2 2 8 2 2], [2 3 5 3 2], and [2 4 4 4 2]. These sequences have identical end values and the same slope (i.e. no overall increase). Indeed, we found a significant preference for flatter sequences for the group and for two of the three monkeys individually. Specifically a bootstrap permutation test showed *m* = 0.35, *p *< 0.004 for the group (subject H: *m* = 0.41, *p *= 0.041; subject J: *m* = 0.30, *p *= 0.0039; subject K exhibited a non-significant trend in the same direction: *m* = 0.18, *p *= 0.081). Note that the preference for flatness within these sequences could be explained by the greater decision weight placed upon later elements in the sequence (see Section 3.4), or possibly by a preference for spreading (Loewenstein & Prelec, 1993).

One possible explanation for the impact of peak values on human retrospective evaluations of sequences is that peak values are over-weighted in memory (Rozin, Rozin, & Goldberg, 2004). Such memory effects may be less important in our task, where the decision occurs less than a second after the end of the sequence. If so, then increasing the working memory load of the task might increase relative preference for peaked sequences. To test this idea, we compared preferences for the same three sequences as above ([2 2 8 2 2], [2 3 5 3 2], [2 4 4 4 2]) in a weak working memory challenge condition: the same task but with a 4 s delay between the end of the last reward in the probe and the choice (Fig. 6B).

We found two effects of delay on preference. First, adding a delay decreased overall preferences for the probe sequence. Second, and more relevant to our hypothesis, monkeys showed a significant preference for the peaked sequence over the other two (bootstrap *t*-test, *p *= 0.001 for the group of animals, and *p *< 0.05 for each animal individually). We observed no difference between preference for [2 3 5 3 2] and [2 4 4 4 2] for either the group or the individuals (*p *= 0.84 for the group and *p *> 0.05 for each individual). These results endorse the memory hypothesis of peak preferences, and suggest that peak- and trend- preferences may be experimentally dissociable.

## 4 Discussion

We used a novel decision-making task to study how monkeys evaluate sequences of rewards. We found that monkeys place more weight on items that occur later in a sequence. These results mirror similar human results showing biases for increasing sequences, and, like them, challenge the validity of simple discounting models of intertemporal choice (Frederick, Loewenstein, & O’Donoghue, 2002; Prelec & Loewenstein, 1991). Models of discounting indicate that monkeys devalue future rewards quite steeply and predict, in direct opposition to our data, that monkeys should strongly prefer decreasing sequences (Glimcher et al., 2007; Kim et al., 2008). These contradictory findings are unlikely to reflect individual differences in our monkeys as they, like most monkeys, exhibit steep discounting as measured in standard intertemporal choice tasks (Blanchard et al., 2013). Our data also indicate that monkeys prefer sequences with prominent peak values, at least when working memory is challenged. We know of no study in humans or animals investigating the effects of working memory challenge on peak preferences. Together, these findings open the window to a deeper understanding of the underlying mechanisms causing the peak bias.

One earlier study examined the sequence preference patterns of rhesus monkeys (Xu et al., 2011). The major finding of this study was that monkeys preferred decreasing sequences of rewards, a finding that is directly opposite of what we report here. We suspect the difference between studies is due to factors of task design. Perhaps the largest difference is the number of trials. That study used 30 trials per day across three days for each of three monkey subjects in the critical experiment (with an additional 10 familiarization trials), whereas we used at least 1000 training trials before we began collecting data. It is possible that monkeys in that study failed to appreciate that choice of the smaller reward would lead to a larger reward later and thus used a larger-sooner heuristic that would have extinguished with more training. Indeed, we regularly observe random or biased preferences during the first hundred or so trials of training of all our tasks, and renewed biases for the first few dozen trials each day. Consistent with this idea, the authors of that study performed four control experiments and found somewhat inconsistent results (effects in only two of the three monkeys, twice significant in the opposite direction in the third monkey) in all of them. For example, experiment 4 in that study, which sought to replicate Experiment 1 with different rewards, found an end bias in one of the three subjects. Moreover, Experiments 2 and 3, which asked subjects to choose between pairs and singletons showed that one of the monkeys (different monkeys in 2 and 3) did not prefer the pair, suggesting a possible failure to understand the task. Various other differences in the studies may have helped to produce these different results. For example, it is possible that the use of a repetition design and the use of five rewards instead of two led to a larger working memory demand, and this elicited the bias (which some have suggested is linked to memory; Fredrickson & Kahneman, 1993). Their task also included a greater inter-reward interval (4 s vs 0.5 s), which may have made the causal relationship between the rewards more difficult to learn. They also used rewards that differed in quality (and thus along multiple dimensions simultaneously) instead of quantity, which also may have made the task more difficult to learn. Finally, as our task was automated and had a much greater number of trials, monkeys may have perceived the task environment as more stable than they did in the Xu study. As perceived stability of the environment can influence temporal preferences in other contexts (e.g. Kidd, Palmeri, & Aslin, 2013), it is plausible that the biases we observe are sensitive to perceptions of environmental stability. It is notable that the design of the Xu et al. is much more similar to that of a standard intertemporal choice task, and that their results are consistent with delay discounting experiments.

### 4.1 General Implications

Our results show that, like humans, monkeys prefer sequences with increasing values. Are the human and monkey biases due to a common cause, or are the two facts coincidental? Our data cannot answer this question definitively. Indeed, human sequence preference effects, which are as diverse as they are ubiquitous, cannot be assigned to the same causes with any certainty. For example, most people prefer that their salary increase over their lifetime (a preference that is not wealth maximizing) and also prefer unpleasant noise bursts that diminish rather than grow over the timescale of seconds to minutes (Loewenstein & Prelec, 1993). These preferences may stem from a single cause or may reflect differing impulses with common results. Thus, we do not have a great understanding of the reasons for human preferences for increasing sequences. We therefore think it premature to argue that monkey peak-end rule biases are the same as those observed in humans, but instead simply that this class of preferences should added to the list of heretofore human-specific biases.

This does not mean we are entirely blind to the cognitive factors that influence sequence biases in our task. In the case of the present study, two factors appear to be particularly important. First, our results suggest that excessive focus on more recent rewards (even on the timescale of seconds) may cause monkeys to integrate past rewards in a biased manner. Second, it appears that working memory capacity may be a critical limiting factor that causes this bias. Note that because working memory is limited to a few minutes, it is unlikely to account for biases in retrospective evaluations of, say, hour-long colonoscopies six months in the past (Redelmeier & Kahneman, 1996). If these possibilities are borne out by subsequent studies, it would suggest that the key factor motivating choices in this task is not a basic preference for increasing sequences, but a perceptual/learning bias coupled with a preference for larger sequences. To the extent that they support this idea, the present results contribute to an emerging picture of animals as canny decision makers that seek to optimize but have specific bounds to their ability to choose utility maximizing options (Bateson & Kacelnik, 1996; Blanchard et al., 2013; Gigerenzer, 2002; Simon, 1955; Stephens, 2002; Stephens & Anderson, 2001).

An important debate in the literature on preferences for sequences is whether we prefer increasing sequences or sequences with large end values. Although the *peak-end rule* favoured by Kahneman and his colleagues is the best known and most widely cited account for such judgments and choices, competing views have been repeatedly expressed, most notably by Ariely and his colleagues, who stress that the *slope* or *rate-of-change* in addition of other factors is the key determinant of preferences (Ariely, Kahneman, & Loewenstein, 2000). In particular, in a comprehensive analysis of the possible underlying determinants of retrospective pain evaluations, Ariely (1998) showed that importance of the pattern of experience including the direction of change, the gradient of the slope, and the final intensity. His conclusion was that the direction of change (trend), not the peak-end, in pain intensity is the single best predictor of retrospective evaluations (for similar evidence in the domain of satisfaction see Hsee & Abelson, 1991; Hsee et al., 1991). However, there remains a debate about the relative contribution of *trend* (defined as direction of change – worsening vs. improving) and the *end* specifically as determinants of preferences. We feel this to be fuelled in part by the flexibility of the peak-end rule – it describes a tendency for respondents to be influenced primarily by these two characteristics (peak intensity and end intensity), but does not specify that they must be weighed equally (indeed, there have been reported examples of cases in which the end was more important, and examples in which the peak was more important, as in Fredrickson, 2000, p. 588). In line with previous work, our data are more consistent with preferences for improving sequences (Loewenstein & Prelec, 1993), because: (1) the greatest increment to preference occurs when the most valued element of the sequence moves from the first to the second position; (2) moving the most valued element to the end has no special effect (and a weaker effect if anything) than moving it later at some other point in the sequence (i.e., this is evidence against an specific ‘end effect’); (3) we detect a peak effect only with a memory challenge.

Another way of looking at our results is to consider the similarities between sequences of rewards and multidimensional rewards. It is well-established that humans have difficulty integrating different dimensions of options to calculate a single utility value (Tversky, 1972). Instead, we generally use heuristic shortcuts, prioritizing certain dimensions (Kahneman, Slovic, & Tversky, 1982). In our task, each step in the sequence is independent and thus in some ways a different dimension. Here we find that monkeys prioritize the more recent rewards in evaluating sequences of options, suggesting that they, like humans, prioritize certain dimensions, perhaps to reduce cognitive load. As far as we are aware, monkeys’ proclivity for dimensional prioritization in standard multi-attribute choice tasks remains unstudied.

### 4.2 Challenge to temporal discounting models

Animal psychologists have long used delay discounting tasks to investigate intertemporal preferences (Ainslie, 1975; Green, Fristoe, & Myerson, 1994; Mazur, 1987; McDiarmid & Rilling, 1965; Rachlin, 2004). It has been repeatedly shown that animals prefer smaller sooner rewards to larger later ones, and that preferences can be described by a hyperbolic (or sometimes exponential) decay curve (Kim et al., 2008; Mazur, 1987). Typically, animals are found to discount half of a reward’s value in 1–5 s (Stephens & Anderson, 2001). These results apply across multiple taxa, including rhesus monkeys, apes, pigeons, rats and even guppies (Mühlhoff, Stevens, & Reader, 2011; Rosati, Stevens, Hare, & Hauser, 2007).

The results of the present study cannot be explained using discounting approaches. Monkeys’ preferences for increasing sequences would require negative discount factors, which have never been observed in the animal kingdom. This possibility is unlikely because our monkeys exhibit garden-variety positive discount factors as measured by standard intertemporal choice tasks (Blanchard et al., 2013). Negative discount factors would also make no evolutionary sense because they would lead to a preference for infinite waiting.

We see three possible interpretations for this discrepancy between intertemporal choice preferences and sequence preferences. First, monkeys may discount strongly in our reward repeat task, but other factors may outweigh the steep discounting. If so, this would suggest that discounting can be easily outweighed and counteracted by other psychological biases, and would challenge its generality and thus its utility as a metric for preferences (Pearson et al., 2010). Second, monkeys’ steep discounting may be limited to certain contexts, such as the intertemporal choice task including single outcomes, and lack predictive validity in other contexts (Stephens & Anderson, 2001). Third, specific design factors in intertemporal choice tasks may provide a misleadingly large reading of animals true discount factors, and animals do not actually discount rewards substantially on the order of seconds.

We favor the third explanation. We and others have previously argued that animals exhibit a bound on their ability to process post-reward delays in delay discounting tasks, and this bound may explain the apparent extreme discounting values often found using these tasks (Blanchard et al., 2013; Pearson et al., 2010; Stephens, 2002). Moreover, animals fail to show steep discounting in naturalistic foraging-like tasks, challenging the validity of intertemporal choice measures (Stephens & Anderson, 2001; Stevens & Stephens, 2008). The present study, which unlike intertemporal choice tasks uses an experiential cue, will necessarily reduce any ambiguity about post-reward delays, and thus provides additional evidence that discount factors lack external validity.

### 4.3 Future directions

The present results raise several important questions. How general are the patterns we observe here? Are they fully explainable due to limited memory or are other effects important? How similar are they do the effects observed in humans? What factors cause these biases and what factors cause the reverse? Do working memory effects in our task relate to putative long-term memory effects in similar tasks with longer delays? We only tested for end-biases with a short delay (0.5 s) between the initial probe presentation and choice, but it is possible that a longer one may elicit a different pattern of biases. Indeed, different delay lengths have been found to affect recall biases in animal working memory tasks (Wright, Santiago, & Sands, 1984). Future work could investigate if this pattern holds true in preference tasks such as ours as well. It remains unclear whether these results will extend to more complex types of rewards, such as intrinsically rewarding sensory cues (Blatter & Schultz, 2006; Deaner, Khera, & Platt, 2005; Watson & Platt, 2012) or longer-duration experiences. Finally, we are curious to know what neural processes lead to these particular patterns of preferences for rewards.

## Appendix A Basic methods

In each session, the animal was transported from the colony at the University of Rochester to the testing room, about 100 feet away in the same building. The testing room was built specifically for primate studies and houses a computer screen and floor plate for firm mounting of the ergonomically designed primate chair (Crist). Animals made all task-relevant decisions using gaze shifts to selected targets. Horizontal and vertical eye positions were sampled at 1000 Hz by an infrared eye-monitoring camera system (SR Research, Osgoode, ON). Stimuli were controlled by a computer running Matlab (Mathworks, Natick, MA) with Psychtoolbox (Brainard, 1997) and Eyelink Toolbox (Cornelissen, Peters, & Palmer, 2002).

A standard solenoid valve controlled the duration of water delivery (Parker). We measured fluid volumes associated with solenoid open time in order to ensure that fluid amounts were linearly proportional to the values instructed by the program. We confirmed that water delivery volume was constant regardless of the volume of water in the reservoir over the ranges used in this experiment. Fluid access was controlled outside of experimental sessions.
